# Impact of environmental inputs on reverse-engineering approach to network structures

**DOI:** 10.1186/1752-0509-3-113

**Published:** 2009-12-04

**Authors:** Jianhua Wu, James L Sinfield, Vicky Buchanan-Wollaston, Jianfeng Feng

**Affiliations:** 1 Department of Neuroscience, Columbia University, New York, NY, 10032, USA; 2 Department of Computer Science, Warwick University, Coventry, CV4 7AL, UK; 3 Warwick HRI, University of Warwick, Wellesbourne, Warwick, CV35 9EF, UK; 4 Centre for Computational System Biology, Fudan University, Shanghai, PR China

## Abstract

**Background:**

Uncovering complex network structures from a biological system is one of the main topic in system biology. The network structures can be inferred by the dynamical Bayesian network or Granger causality, but neither techniques have seriously taken into account the impact of environmental inputs.

**Results:**

With considerations of natural rhythmic dynamics of biological data, we propose a system biology approach to reveal the impact of environmental inputs on network structures. We first represent the environmental inputs by a harmonic oscillator and combine them with Granger causality to identify environmental inputs and then uncover the causal network structures. We also generalize it to multiple harmonic oscillators to represent various exogenous influences. This system approach is extensively tested with toy models and successfully applied to a real biological network of microarray data of the flowering genes of the model plant Arabidopsis Thaliana. The aim is to identify those genes that are directly affected by the presence of the sunlight and uncover the interactive network structures associating with flowering metabolism.

**Conclusion:**

We demonstrate that environmental inputs are crucial for correctly inferring network structures. Harmonic causal method is proved to be a powerful technique to detect environment inputs and uncover network structures, especially when the biological data exhibit periodic oscillations.

## Background

One of the main topics in system biology is to uncover the complex network structures in a biological system [1,2]. In comparison with simple systems, nowadays the researchers always face larger and more complex dynamic interactive systems (e.g., neural networks and gene networks). Traditional techniques, such as the cross-correlation and partial coherence analysis [3-7], are inadequate to clearly and explicitly reveal the true network structures for such a complex system. These techniques neither take time dimension into consideration nor reveal the directional interactions, thus they cannot configure a dynamic interactive system with time. Over the past few decades several advanced techniques such as dynamic Bayesian networks [8] and Granger causality [9-13] have been developed to identify network structures in dynamic systems. Granger causality only reveals direct causality between pairwise units with linear interactions, thus conditional and partial Granger causality [14,15] and kernel Granger causality [16] have been proposed to deal with indirect causal interactions among multiple variables.

In multivariable (more than two) situations, one time series can be connected to another time series in a direct or an indirect manner, raising the important question of whether there exists a (direct) causal influence between two time series when the influence of other time series are taken into account. In such cases, repeated bivariate analysis can be misleading. For example, one time series may falsely appear to cause another if they are both influenced by a third time series but with different time delays. The conditional Granger causality [14,15] aim to deal with the causal interactions among multiple variables. However, the applicability of the kernel Granger causality or the conditional Granger causality largely depends on the experimental ability to measure all relevant variables in the system, but it is usually not feasible in the biological recordings. Environmental inputs, including exogenous inputs from external sources and unmeasured endogenous variables, cannot be all captured by experimental techniques, but such environmental inputs can confound the accuracy of causal influences and thus degrade the credibility of the uncovered network structure. For example, in our experimental data recorded from the inferotemporal (IT) cortex of sheep, every measured neuron receives common exogenous inputs from the visual cortex and feedbacks from the prefrontal cortex [7,15]. Even with advanced multielectrode array (MEA) techniques, it is only able to record a tiny subset of interacting neurons in a single area [15,17] and there are bound to be endogenous variables. Hence controlling environmental inputs is a critical issue when applying Granger causality to experimental data. Recently, partial Granger causality [15,18] is developed to eliminate the influences of exogenous inputs and latent variables, but a full elimination is only possible if all common inputs have a more or less identical influence on all measured variables. It is generally not realistic that all measured variables can receive an almost identical and common influence in experimental recordings. In fact, the common influence degrades due to spatial increment. In such cases, it is critical to identify which measured variables received environmental inputs, and what is the impact of the environmental inputs on configuring network structures?

We take a system biology approach to answer the questions above. Most current techniques largely ignore the natural dynamical characteristics of the biological data, which usually exhibits highly rhythmic (periodic) oscillations, especially under periodic environmental influence, e.g. light-dark condition [19-23]. Such natural periodic dynamics of experimental data can provide important information in model fitting and error estimation. To overcome the limitations of current causality techniques and make full use of harmonic oscillation characteristic of experimental data, we consider a harmonic oscillator, or a set of harmonic oscillators, to represent the environmental inputs. The harmonic oscillators can be mathematically formulated by the hidden periodic model [24,25]. We extend the current linear Granger causality model (Autoregressive model) by inclusion of the typical harmonic oscillators embedded in the experimental recordings. If the inclusion of harmonic oscillators can significantly reduce the variance of the prediction error, then the environmental causal influence can be reduced or eliminated. The mathematical representation of a harmonic oscillator model is derived in Method section and the application of the harmonic Granger causality approach is elucidated in Result section. Although the techniques of Granger causality are based on time series data, additional useful information can be revealed when the analysis is performed in the frequency domain [14,18,26,27]. Investigating the causal interactions between different frequencies adds another dimension to the already complex identification of spatiotemporal and frequency-specific rhythmic oscillations. Conventional cross-frequency interactions are characterized by the synchrony of phase, recognized as 'n:m phase synchrony' [28,29]. Phase synchrony indicates amplitude-independent phase-locking of n cycles of one oscillation to m cycles of another oscillation, however, this method largely ignores the information carried by the amplitude and the coupling effects between phase and amplitude. Importantly, phase information can be sensitive to random noise [30], while in most experimental data the true signal is heavily masked by random noise. In this study, as a by-product, we also assess whether it is reliable to use the phase information between two (oscillating) units to estimate the causality. Our simulation results clearly demonstrate that it can be very insufficient and inaccurate to use only the phase information to characterize a causal interaction, but the approach developed in the current paper works. We first apply the harmonic oscillator idea to a toy model and validate it by comparing with the conventional Granger causality. Then we investigate the effect of multiple oscillators by employing a small sparsely connected network. Finally we apply the harmonic Granger causality to a real biological network of microarray data of the flowering genes of the model plant Arabidopsis Thaliana. We aim to identify which genes are directly affected by the presence of the sunlight, and uncover the causal interactions among genes. Although tens of thousands of known genes within Arabidopsis are collected with Microarray, only those genes known to be involved in the flowering of the plant (8 genes in this case) are analyzed by our harmonic Granger causality. The method successfully reveals the genes that most possibly receive environmental inputs. We finally compare our causal network with other candidate models in the literatures [31-33]. With this system biology approach, our causal network depicts all possible connections reported in the literatures [32,33], and also reveals two more connections that do not exist in the known candidate models.

## Results

### Toy Model & Validation

In this simple toy-model example, we compare the performance of traditional Granger causality and harmonic Granger causality on four simple model configurations. We show that the traditional Granger Causality analysis is not sufficient to describe the influence of one time series upon another in the presence of an external driving force. The full simulated model under consideration is described as follows:(1)

Within this model we consider four configurations, firstly where both node *X*_*t *_and *Y*_*t *_experience an environmental input, secondly where *X*_*t *_experiences the environmental input and *Y*_*t *_does not, thirdly where *Y*_*t *_experiences the environmental input and *X*_*t *_does not, and lastly where neither *X*_*t *_or *Y*_*t *_undergo the influence from the external driving oscillation. Despite the external driving oscillation exerted on either *X *or *Y*, there is a coupling frequency of 34 Hz between *X *and *Y *for all four configurations. In each of the simulations, *f*_*x *_and *f*_*y *_are equal to 10 *Hz*, the input phases, *ϕ*_*x *_and *ϕ*_*y *_are set to zero, and the variances of inherent noises, *ε*_*t *_and *η*_*t*_, are set to 0.1. In all the following simulations, the values of *C*_*x *_and *C*_*y *_are 0.07 and 0.05, respectively.

**Figure 1:**
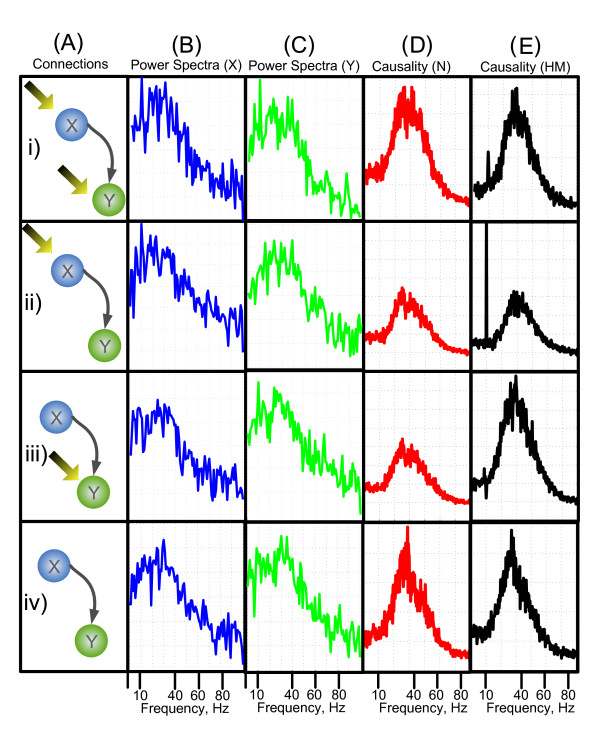
**Validation of the hidden harmonic methodology**. Four different model configurations are considered. In each configuration node X has a causal influence of node Y. In (i) both of the nodes have environmental inputs, in (ii) and (iii) the simulation has just node X or node Y have an external input, in (iv) neither X or Y have external inputs. (A) shows the connection configurations. In (B) and (C) the power spectra of each configuration are shown in frequency domain, node X is shown in blue, node Y is shown in green. (D) and (E) show the causality spectra from X to Y, *f*_*X*→*Y*_, the causality calculated using the normal Granger AR method is shown in column (D), the harmonic causal method is shown in column (E).

In the first case where both nodes experience the environmental inputs, Fig. 1Bi and 1Ci shows that both *X*(*ω*) and *Y*(*ω*) have components at the driving oscillation frequency of 10 *Hz*. The causal link known to be present from X to Y should mean that there is a spike in the causality spectra at the driving frequency. Fig. (1Di) and (1Ei) show that the normal Granger AR method can only detect the causality spectra at the intrinsic coupling frequency around 34 Hz, but the Harmonic method is capable of detecting the causal influence at the external driving frequency at 10 Hz. The reason is that the driving force from *X *to *Y *comes from the factor 0.3*X*_*t *_- 2 (Eq. 1), which contains both intrinsic coupling frequency and external driving frequency. Harmonic method can better fit and predict the data at external driving frequency such that it can detect the external driving frequency at 10 Hz better than normal Granger AR method.

The second case is where *X*_*t *_receives the driving input and *Y*_*t *_does not. Fig. 1Bii shows that the peak in the spectra can be seen in the X-channel at 10 *Hz *representing the external driving oscillation, and although there is no inherent 10 *Hz *oscillation in the *Y*_*t *_channel, the spectra of *Y *(*ω*) (Fig. 1Cii) shows that there is an induced oscillation at this frequency, hence one would expect that the causality of *F*_*X*→*Y *_will show a large component at the driving frequency. Fig. (1Dii) and 1Eii show the causality, *F*_*X*→*Y *_calculated using both AR and harmonic methods. The AR method shows little peak at the driving frequency, while the harmonic method produces a large spike in the causality at the frequency 10 Hz. In third case where *Y*_*t *_receives the external input, Fig. (1Ciii) shows that the peak in the spectra can be seen in the Y-channel at 10 *Hz *representing the external driving oscillation. We would expect that in this case the causality shows no peak at the driving frequency as *X*_*t *_contains no driving oscillation: Fig. (1Diii) and (1Eiii) show the causality, *F*_*X*→*Y *_calculated using AR (panel *D*_*iii*_) and harmonic methods (panel *E*_*iii*_). In this model configuration, both the AR and the harmonic methods produce similar results. However there is a drastic decrement in the spectra of the harmonic method at the driving frequency in the causality, qualitatively, this cannot be the case, so in this instance it is preferable to use the AR method to calculate the causality spectra as *X*_*t *_contains no driving oscillation. The causality decrement near the driving frequency may be caused by the inclusion of the harmonic term, which may extract the intrinsic power of the signal *Y *at the driving frequency of 10 Hz. The decrement means that signal *Y *was mainly driven by the harmonic term at 10 Hz and signal *X *did not contribute much at this frequency. For other frequency range, harmonic term did not influence signal *Y *and the driving force mainly came from signal *X*. The final model configuration considered is where neither *X*_*t *_or *Y*_*t *_has the driving input. Fig. (1Div) and (1Eiv) show that in this case there is very little difference in the causality spectra obtained using the two methods.

Through this simple toy model we have demonstrated that the normal Granger Causality in the frequency domain is not sufficient to detect interactions at all frequencies in the presence of an environmental input. Furthermore we have demonstrated that the causal method with the additional harmonic term produces more consistent and accurate results than the traditional Granger causality method at most instances. The harmonic causal method and the traditional Granger causality method can be a good complement to each other when applying to the time series with or without oscillatory environmental influences.

### Investigating the Effects of Phase

It is a common scenario in physics to determine the driving relationship between two oscillators. The phases of the two oscillators can be interleaved throughout the time, thus phase may not be an accurate indication of the causal interactions between the two oscillators. One of the main motivations of introducing the harmonic term is to investigate the effects of phase and to determine whether it can be used as an indication of the amount of causal interactions between oscillatory signals. Consider again the case where *X*_*t *_drives *Y*_*t*_, Eq. 1 shows that the influence of the time series *X*_*t *_upon *Y*_*t *_is encapsulated in the oscillatory and noise terms. However, the amplitude of the resultant oscillation will depend on the phase difference of the two harmonic terms. This can be seen by considering the addition of the two oscillatory terms. Let *O*_*y *_= *C*_*y*_*cos*(2*πf*_*y*_*t *+ *ϕy*) and the harmonic component from *X*_*t *_be , then in the case that the frequency of the oscillations are equal, *f*_*x *_= *f*_*y *_= *f*, the two oscillation terms can be combined as follows:(2)

where(3)

and(4)

It is able to analyze the effect of input phase upon the level of influence of one time series upon another. Eq. 3 reveals that the magnitude of the resultant harmonic term is indeed a function of both  and *ϕ*_*y*_. The oscillating term, , oscillates sinusoidally and can take values in the range [-1, 1].

The relative effect of this oscillation upon  depends on the values of *C*_*y *_and . The extremes of  are given by , and it can be shown that the maximum effect of the phase differences happens when *C*_*y *_=  and the minimal difference happens when either *C*_*y *_or  equals zero.

**Figure 2:**
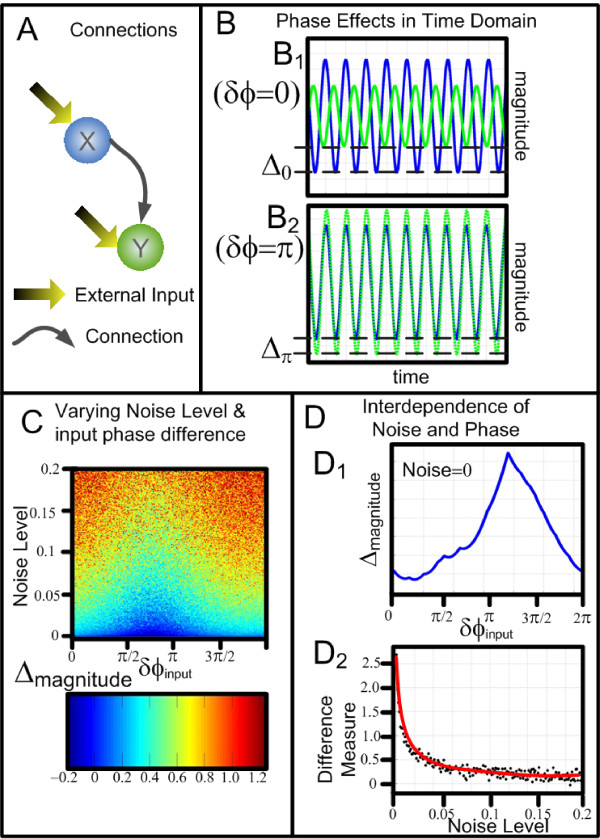
**The Effects of Varying Input Phase**. A): shows the model configuration where X has a causal influence upon Y and there is no feedback. Both X an Y have an external, environmental oscillatory input. B): shows approximately 1 sec of simulated time domain plots the blue trace is *X*_*t*_, the green trace is *Y*_*t*_. In *B*_1 _*ϕ*_*x *_= *ϕ*_*y *_and in *B*_2 _*δϕ *= *ϕ*_*x*_- *ϕ*_*y *_= *π*, in both *B*_1 _and *B*_2 _the X trace is identical, yet the trace of Y is shown to be greatly changed simply by altering the phase difference. The difference in the magnitude is denoted by Δ. As the inherent noise will mask the effect of the phase differences, C) shows the effect on Δ by varying both input noise and *δϕ*. *D*_1 _shows how this Δ changes by varying *δϕ *in the absence of noise. *D*_2 _shows how the effect of noise in the system lessens the effect of the phase differences.

In order to demonstrate the effects of phase changes, the same model as Eq. 1 is used. Fig. 2 shows the enormous consequences of the magnitude of the resultant signal by simply varying the phase of the environmental input, *ϕ*_*x *_and *ϕ*_*y*_. Fig. 2A shows that the configuration of the model with both nodes of the system receiving external environmental inputs and a causal link from X to Y. Fig. 2B shows two examples in the time domain traces obtained in the absence of environmental noise. The upper panel Fig. 2B1 shows the time domain traces when the phase difference of the external inputs is zero (*ϕ*_*x *_= *ϕ*_*y*_) and the lower panel Fig. 2B2 shows the scenario when *ϕ*_*x *_- *ϕ*_*y *_= *π*. In each of these diagrams the blue trace, which is the node *X*_*t*_, is unchanged, however the time series associated with *Y*_*t *_change considerably. The difference of the maximum amplitudes of the two time series is denoted by Δ. The colourmap figure shown in Fig. 2C plotted the value of Δ against the values of the input noise and the input phase differences. It can be seen that the effects of the phase differences is lessened by increasing the noise in the system. Fig. 2D reveals the interdependence between noise and phase in this system. The upper panel Fig. 2D1 shows the amplitude difference against the phase differences in the absence of noise. The reason why the peak does not happen at *p *is because *C*_*x *_and *C*_*y *_are different, and there is a phase difference. If *C*_*x *_= *C*_*y*_, then the peak would be exactly at *p*. The lower panel Fig. 2D2 shows how a measure of the difference varies with increasing noise. The measure of the difference is defined as . As the noise increases the measure difference tends to zero, confirming that the noise levels can mask the phase effects.

**Figure 3:**
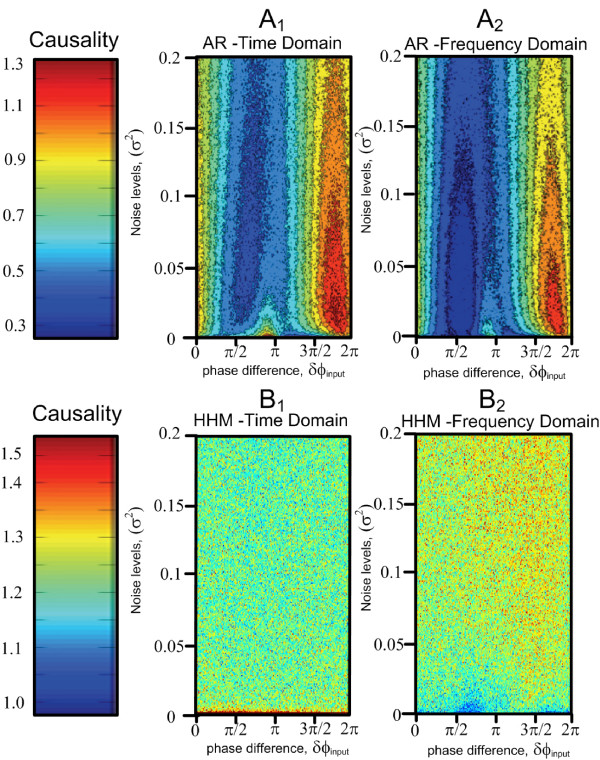
**Each colourmap shows the causality calculated whilst varying noise and input phase difference**. *A*_1 _shows the causality calculated using the AR method in the time domain, *A*_2 _shows the causality calculated by integration of the AR method frequency domain causality. *B*_1 _shows the causality calculated using the harmonic method in the time domain, *B*_2 _shows the causality calculated by integration of the harmonic method frequency domain causality.

We have shown that the phase of the external driving oscillation has an effect on the amplitudes of the resultant time series, then the real question is if this effect can be detected as a difference in the level of causality. Recall that the actual level of causal interaction is not varying and the influence that *X*_*t *_exerts over *Y*_*t *_does not alter throughout the simulations. The investigation into the effects of phase continued with a series of calculations determining the level of causality. Fig. 3 shows the causality detected in the system obtained by four different methods. Fig. 3A1 shows the time domain causality detected using the normal Granger method, Fig. 3B1 shows the time domain causality detected using the harmonic method, Fig. 3A2 and 3B2 show the causality detected using the AR and harmonic methods in the frequency domain. For consistency we require that:(6)

where *F*_*X*→*Y *_is the causality on the time domain and *f*_*X*→*Y *_(*ω*) is the causality at frequency *ω*. Eq. 6 is the Kolmogorov condition that indicates the equivalence of the causality in time and frequency domain.

Comparing the AR and harmonic causality results, the amplitude differences influenced by the phase differences are represented in the AR method but not in the harmonic causality results, which imply that the harmonic approach must be used for a true indication of the level of causal interaction as it is robust in presence of the external driving oscillation.

Comparison of Fig. (3A1) and Fig. (3A2) shows that the Eq. 6 holds for the AR method and there is a high level of correspondence between the causality calculated in the time domain and frequency domain. Similarly, comparison of Fig. (3B1) and Fig. (3B2) shows that to a large extent the harmonic method is consistent with the Kolmogorov condition in Eq. 6. The causality calculated by harmonic method does not depend on the phase difference no matter it is in the time domain or frequency domain.

**Figure 4:**
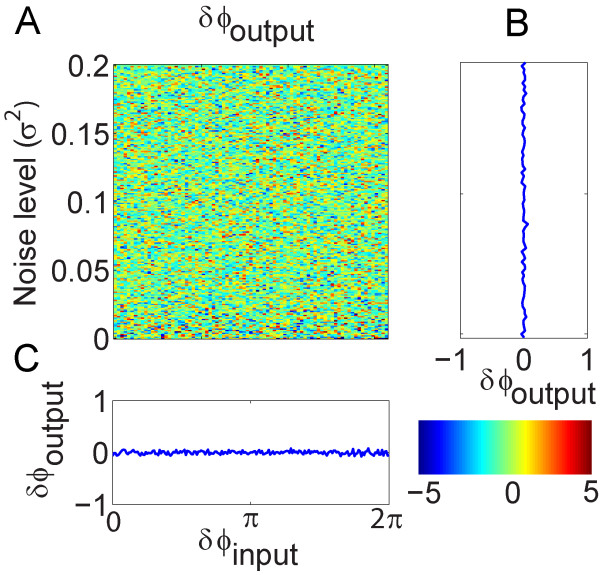
**Investigation of the interrelation between the phase difference of input and output signals and noise level**. A): a colourmap plot of the phase difference of output signal against the phase difference of input signal and noise level. The phase difference of output signal is almost uniformly distributed for varying noise level and the phase difference of the input signal. B) and C) demonstrate the averaged phase difference of the output signal against noise level and the phase difference of the input signal, respectively.

We further investigated the relationship between the phase difference of input and output signals, and the influence of noise level. The same configuration model presented in Fig. 2A was used to demonstrate this interrelation. The phase difference between two input signals varied from 0 to 2*p *and the noise level (variance of the white noise) increased from 0 to 0.2. The phase difference of signal *X *and *Y *was plotted as a function against the phase difference of the input signals and the noise level. Fig. 4A shows the colourmap of the interrelation. The color intensity represents the phase difference for the output signals. Fig. 4B and Fig. 4C show the averaged intensity along noise level and the input phase difference, respectively. It is clear that the phase difference of the output signals does not depend on the phase difference of the input signals and the noise level. The results indicate that the phase information cannot be used alone to accurately determine the causal relationship between any two signals. The interpretation of the causality based on phase should be cautious as the causality may not reflect the true relationship.

### Investigating Effect of Multiple Oscillators

**Figure 5:**
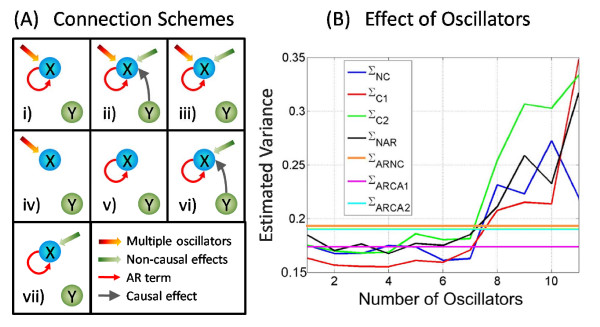
**Schematic connection plots and estimated variance for different number of oscillators**. A): Seven connection schemes for causal and non-causal influence when there are or not harmonic oscillators. B): Using a simple small sparsely connected network consisting 5 nodes. The number of oscillators was increased and various fitting algorithms are applied.

In experimental recordings, the measured variables are usually influenced by many environmental inputs, thus multiple oscillators have to be considered. In order to reveal the power and limitations associated with the additional oscillators, a simple system was considered and the errors of seven different connection schemes was compared. The schematic plot for seven connection schemes is displayed in Fig. 5A, and the error terms corresponding to each schemes are described as follows:

i) Σ_*NC*_: AR + harmonic oscillations, no causality

ii) Σ_*C*1_: AR + harmonic oscillations, influence from causal node

iii) Σ_*C*2_: AR + harmonic oscillations, influence from non-causal nodes

iv) Σ_*NAR*_: harmonic oscillations only

v) Σ_*ARNC*_: AR only

vi) Σ_*ARCA*1_: AR, influence from causal node

vii) Σ_*ARCA*2_: AR, influence from non-causal nodes

To investigate the effect of multiple oscillators on the goodness of fit, we consider a simple network consisting of five nodes in various random configurations. The variances are estimated for each of the connection scheme in Fig. 5A, and the number of harmonic oscillators varies from 1 to 11. The results of this simulation are shown in Fig. 5B. Inspection of Fig. 5B reveals some of the issues involved in using the harmonic oscillators to provide a full description of the time domain evolution. Firstly looking at those variance estimates with no harmonic oscillatory components (Σ_*ARNC*_, Σ_*ARCA*1 _and Σ_*ARCA*2_), as expected in Fig. 5B that these are constants for increasing numbers of oscillators (invariant as they are independent of oscillators). The estimates of Σ_*ARNC *_and Σ_*ARCA*2 _are very similar, because the non-causal nodes do not provide useful information for fitting the target node. The causal estimate (Σ_*ARCA*1_) is the best estimate when no harmonic oscillators are considered.

Inspection of estimates in which oscillations are included reveals an important trend; one would expect that as the number of oscillators increases, the estimates become more and more accurate. Theoretically speaking this should be the case, however realistically it is shown not to be the case as in Fig. 5B. When the number of oscillators goes beyond seven, the estimated variances drastically increase. The reason for this situation is the overfitting problem. The total number of parameters needed to be estimated depends on the number of oscillators (three extra parameters per oscillator), and the known data cannot fit the model if the parameters go beyond the amount of data. As expected, the causal estimate (Σ_*C*1_) provides the best estimate for small number of oscillators (minimum at oscillators = 3). This simulation reveals that the number of oscillators does not exceed certain value (seven in this case) if we do indeed obtain a good estimate far surpassing the accuracy of the estimate using only AR. We have to perform goodness of fit test to determine the number of oscillators that can help to fit the data best.

### Gene Data & Network

**Figure 6:**
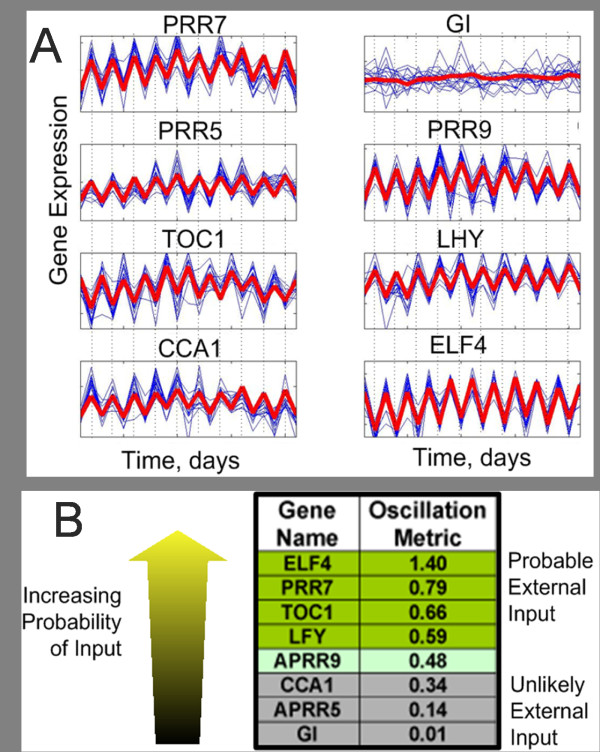
**Time domain traces of gene expression of eight genes under scrutiny and possible environment input**. A): The time domain plots, in blue is the 16 repetitions of the experimental data and in red is the parameterized fitted data (Causal harmonic fitting). With the exception of the gene GI, each of the gene exhibits high periodicity and high levels of repeatability. B): The 8 genes and the oscillation metric, those with a larger metric are more likely to contain an external oscillatory input.

Having shown the necessity of applying the harmonic approach to identifying causality in data sets where it is known that an external environmental oscillation is driving the time series, it is necessary to apply this system approach to real experimental data. The microarray gene data pertaining to the flowering clock cycle of the Arabidopsis is one such example where this methodology may prove enlightening. The plants (Arabidopsis) are grown in laboratory conditions, where they are subjected to 12 hours of artificial daylight followed by 12 hours of no light representing night time. Gene microarray data is collected at regular intervals (twice a day) throughout the experiment. Inspection of the time series of this gene expression data reveals that there is a clear periodic oscillation which corresponds to day/night time periods, suggesting that the expression levels of the genes depend upon the amount of sunlight present (see Fig. 6 panel A).

We consider the time domain change of eight genes involved in the flowering system of the Arabidopsis, namely CCA1, ELF4, GI, LHY, PRR5, PRR7, PRR9 and TOC1. The time domain trace of the expression of these genes is shown in Fig. 6 panel A. Each of the genes with the exception of GI exhibits highly oscillatory behaviour with period of one day. This periodicity is attributed to the presence of incident sunlight during the day time and its absence during the night. Some of the genes are directly affected by the light and are expressed to a greater or less extent during the day. The experimental data used for this analysis is over a period of 11 days with two measurements per day, hence data for each gene consists of 22 data points and there are 16 repetitions of each time point (4 biological repetitions and 4 technical repetitions for each measurement).

The task regarding this data set is twofold, firstly we wish to identify which of the genes are driven by the external oscillation. And secondly, we wish to determine how the genes are connected to form the causal network governing flowering of the plant. The method to determine environmental input and network connectivity is as follows. There are 56 pairwise combinations possible with eight genes; for each of these 56 gene pairs the parameters of four candidate models were calculated. These models are presented below:

where *M *is the number of genes in the network, *p *is the number of lagged observations used in the model. *p *can be determined by a quantity called Akaike Information Criteria (AIC) [34]. The four candidate models are descriptions of an estimation of *X*_*g *_used to determine the effect of *X*_*h *_with or without the external driver. The variance of the errors associated with each of the models are: Σ_1_, Σ_2_, Σ_3_, Σ_4 _respectively.

The errors (Σ_*i*_) - associated with each model were estimated for each gene pair. Using these errors we can infer both the presence of an external environmental driver and the possibility of a connection between the pair of genes.

Comparison of Σ_1 _and Σ_3 _and comparison of Σ_2 _and Σ_4 _reveal that whether particular gene may have an external input as these estimates differ only by the presence of the oscillatory input. If the estimate is improved appreciably by the addition of the harmonic term, then it is possible that this gene receives an environmental input. Therefore for each gene, a measure of the likelihood of input presence is obtained as follows: . Fig. 6B shows the oscillation metric for each of the 8 genes in the network. This method merely states which of the genes is more likely than others to have an input, so a decision must be made as the value of the oscillation metric is the cut-off point. At a first estimate, the value of *M *= 0.5 has been selected. Figure 6B shows that selecting a cut-off value of 0.5 for the oscillation metric leads to the following genes having an external oscillatory input; ELF4, PRR7, TOC1, LFY.

Having calculated whether the genes have an external input, it is possible to obtain the causality of each pair of genes. Consider the causality between gene *X *and *Y*. If gene *X *has an external input, then the causal influence *X *exerting upon *Y *is: . Whereas there is no external input to gene *X*, then the causality will be given by: . The errors (Σ_*i*_) - and hence the causality associated with each model were found for each pair of genes and then sorted in descending order. Those with the highest level of causality deemed more likely to have a connection.

**Figure 7:**
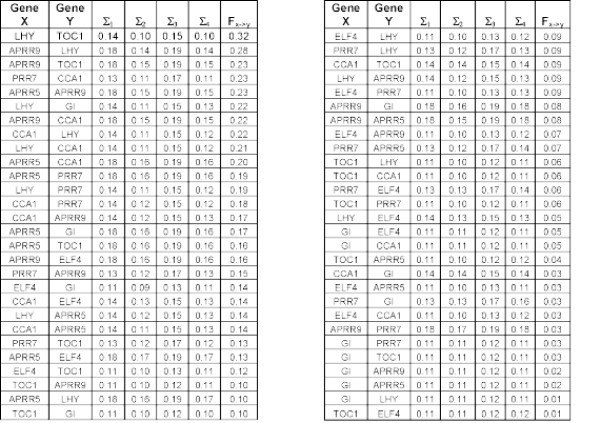
**The errors associated with each of the gene pairs for each of the four candidate models, the causality, *F*_*X*→*Y *_is calculated either with  or  depending upon whether the gene has an external input**.

**Figure 8:**
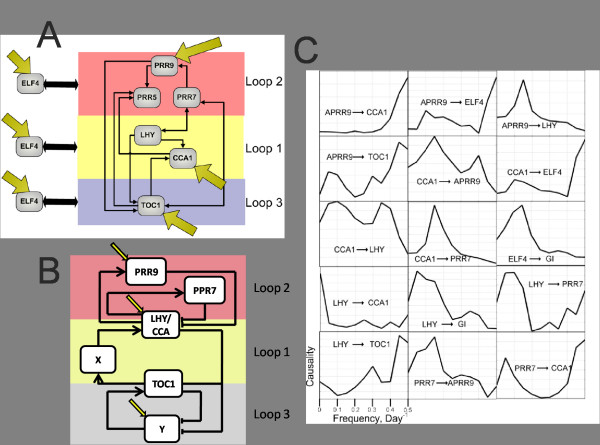
**A): Candidate gene network calculated using the both the harmonic and non-harmonic schemes**. B): reproduced Ueda's candidate model [32,33]. C): Causality spectra for 15 connections inferred from the network.

The table of errors (Σ_*i*_) shown in Fig. 7 is used to find the most likely connection in the gene network. One such candidate network is shown in Fig. 8A.

To validate the gene network generated by harmonic Granger causality, we compare it with other candidate network in the literature of circadian clock cycle by Ueda [31,32]. The candidate network is reproduced in Fig. 8B. The three loop model was first proposed by Locke [31] and then modified by Ueda [32,33]. In Ueda's model two hypothesized unnamed genes are omitted in our model and the genes LHY and CCA1 are treated as one entity. Our analysis reveals that four of the genes in this network receive external inputs: PRR7, CCA1, CCA1 and ELF4. The first two of these agree with Ueda's network. The Ueda's network states that TOC1 does not receive external influence but the hypothesized gene does. It is possible that this influence has been included in the time series of TOC1. The structure of the two networks also are very closely related, both showing a high level of connectivity. Perhaps the biggest difference is that our method shows that there are connections between PRR7/PRR9 and TOC1, while Ueda's model does not reveal such connections.

In addition to finding the likely connections between the genes, the frequency domain analysis allows us to investigate the frequencies at which one gene drives another. Fig. 8C shows the causality spectra calculated for each of the 15 connections believed to exist in the gene network shown in Figure 8. *f*_*X*→*Y *_(*ω*) is shown on frequency domain between [0, 0.5]/day^-1^, whether this is calculated using the harmonic or AR method depends upon whether Gene *X *is thought to have a external input, i.e., those with an external input are calculated with the harmonic method, and those with no external input are calculated using the AR method. It is often the case that the two methods produce very different causality spectra, so selecting the correct method is essential, in many instances the AR method predicts a causal influence at the driving frequency, yet the harmonic method does not.

## Discussion

Although harmonic causal method has greatly improved the performance of causal connection detection, there are several issues that harmonic causal method cannot answer or infer at this stage. First, the application of harmonic Granger causality has a precondition that the signal is influenced by harmonic environmental inputs. Most biological data exhibits harmonic oscillatory behavior, while there may also exist other form of nonlinear exogenous input other than harmonic form. The harmonic method cannot deal with such nonlinear interactions, and its application to nonlinear exerts would depend on specific problems. Second, harmonic causal method is developed to detect the directional causal interactions between any two elements, but it has no ability to determine the influence is positive or negative. For example, one neuron can exert an excitatory or inhibitory effect on another neuron; or one gene can cause another gene's expression level up or down. It is not possible at this stage to figure out the positive or negative effect by only determining the directional causal interactions. Third, one should take extra caution when applying multiple harmonic causal method as the overfitting problem can easily happen. Excessive number of harmonic oscillator will generate inaccurate model estimations and predictions.

## Conclusion

We have presented a system biology approach to study the impact of environmental inputs on recovering network structures. The harmonic modification of the Granger causality is essential if we want to have the complete picture of causal interactions between elements in a system in the presence of a periodic environmental oscillation. The toy model example demonstrated that the conventional Granger causality was not sufficient to reveal the level of causal influence in the presence of an oscillatory driver. Furthermore, the toy model was able to validate the estimates used in the definition of the frequency domain harmonic causality. One of the motivations for the introduction of the driving oscillation was to investigate whether it is feasible to use phase differences between oscillatory signals to assess the causality. We also showed that the apparent level of influence on the conventional Granger causality was tightly related to the phase difference and noise intensity, and this artefact was enough to render the estimation of the conventional Granger causality. The harmonic Granger causality was not sensitive to these phase effects and produces more accurate estimate of the true causality. We also applied the harmonic method to detect external drivers and causal connections in a gene network. We were able to predict which genes receive an environmental input from the sun and these results are in agreement with the experimental results to a large degree. Furthermore, we were able to reproduce the network, which not only reveals known connections but also predicts new connections comparing with classical candidate models.

Our approach clearly demonstrates that by including appropriate environmental (oscillatory) inputs in a conventional reverse-engineering approach could significantly improve its accuracy. Obviously the same idea could be applied to other approaches such as the Bayesian network inferences and information theory approach.

## Methods

### Causality in the Time Domain

In order to infer the connections between the elements of a system constituting a network, we propose an extended Granger Causality whereby a harmonic oscillatory term is added to the normal autoregressive (AR) and error terms of the conventional Granger analysis, and such simple modification can yield surprising and useful results. To appreciate the effect of the proposed modification and the power of the addition of the harmonic oscillation to the Granger causality analysis, we provide the conventional Granger causality in the supplementary material (Additional file 1) and proceed directly to the formulation of harmonic Granger Causality.

Consider two time series *X*_*t *_and *Y*_*t*_, a general form of an autoregressive model with environment inputs (sinusoidal form) has the following vector autoregressive representation:(7)

A joint autoregressive representation having information of past measurements of both time series *X*_*t *_and *Y*_*t *_can be written as(8)

where *p *is the maximum number of lagged observations in the model. *ε*_*it*_, *i *= 1, 2, 3, 4, are prediction errors with variance Σ_*i*_, which are uncorrelated over time. The value of Σ_1 _measures the accuracy of the autoregressive prediction of *X *based on its previous values and the harmonic term, whereas the value of Σ_3 _represents the accuracy of predicting present value of *X *based on previous measurements of both *X *and *Y *and the harmonic term. According to the causality definition of Granger, if the prediction of one process is improved by incorporating past information of the second process, then the second process causes the first process. In other words, if the variance of the prediction error for the first process is reduced by the inclusion of the past histories of the second process then a causal relation from the second process to the first process exists. This causal influence is quantified by(9)

It is clear that *F*_*Y*→*X *_= 0 when there is no causal influence from *Y *to *X *and *F*_*Y*→*X *_> 0 when there is. Similarly, define causal influence from *X *to *Y *as(10)

Due to the natural rhythmic dynamics of the experimental recordings, the environmental inputs (denote as *E*) are represented by the harmonic terms. While the inclusion of the harmonic terms can exclude the periodic influence caused by the environmental inputs, thus the prediction error can be better estimated and truly reflect the interaction between two processes. We can quantify the influence of environmental inputs (*E*) by recalling the joint autoregressive model of *X*_*t *_and *Y*_*t*_.(11)

By definition of Granger causality, the causal influence from *E *to *X *or *Y *can be defined as(12)

### Causality with multiple Oscillators

In many aspects, the addition of a single oscillator is a generalization of the Granger causality, however the application of the adaptation to the normal autoregressive approach is limited to only one external driving force. A further generalization considered here consists of adding more oscillators to the AR model. The interpretation of this approach is as follows: the first and most simple interpretation is that the additional oscillators represent more external oscillatory driving forces, thus being a mere extension of the single oscillator case. A more enlightening and more useful interpretation is that multiple oscillators represent a 'field' of unknown influences upon the network. We know from Fourier theory that any function or signal can be represented by a (possibly infinite) summation of sinusoids, therefore the addition of multiple oscillators in this fashion can, in theory, account for any incident influence upon each of the variables within the system. This interpretation has some rather useful applications. Consider a large sparsely connected network. It is a typical scenario that due to some experimental limitations, we can only record a small proportion of information in the network as a whole. Ideally we wish to reconstruct the structure of the subnetwork for which we have recorded. Hopefully by considering multiple oscillators, this Fourier-like method will provide an avenue to recover the structure of the subnetwork.

By analogy with the single oscillator case, in multiple oscillator scenario there exist a number of unknown external inputs about which we can obtain no information. These unknown inputs and their influences are to be approximated by the summation of many oscillators. In order to calculate the interaction from *Y*_*i *_to *X *with many external oscillators and known variables, we can write the equations as(13)

In Eq. 14, all known variables (*Y*) are included in the AR terms, while in Eq. 13, the variable *Y*_*i *_is excluded in the AR terms. *M *is the number of known variables, *p *is the total number of previous time steps included and *N *is the number of oscillators considered in the estimation. The errors associated with noncausal and causal estimations are *ε*_*nc *_and *ε*_*c *_respectively.

The level of causality from *Y*_*i *_→ *X *is quantified as:(15)

If  then there is no causal influence from *Y*_*i *_to *X*. If F_*Y*→*X *_> 0 then there is a causal influence from *Y*_*i *_to *X*.

### Causality in the Frequency Domain

The key of information extraction is to switch from temporal domain to frequency domain in which their information content can usually become more obvious. Fourier transform provides spectral power that identifies the amplitudes of sine functions of various frequencies that exist throughout the entire duration of the signal. The time domain Granger causality and the harmonic modification can be transformed into the frequency domain, whereby we can obtain the causality spectra showing the frequencies at which the influence of one variable is exerted on another. Expressing the harmonic time series approximations in matrix format leads to the following expression:(16)

where the summation of over the time lags is implied such that . *L *is the lag operator. And the zeroth terms of the coefficient matrix are such that *a*(0) = 1, *b*(0) = 0, *c*(0) = 0 and *d*(0) = 1. *O*_*x *_and *O*_*y*_are the harmonic terms. Take the Fourier transform on both sides of this matrix equation and then multiply by the inverse of the matrix, then express *X*(*ω*) and *Y*(*ω*) in terms of the error and harmonic oscillations, we can obtain the transfer function:(17)

Now the spectra of *X*(*ω*) and *Y *(*ω*) can be can be derived as(18)

Thus the spectra are given by:(19)

It is instructive to investigate the components which constitute the spectra of *X*(*ω*) and *Y*(*ω*). Expanding the expression for *S*_*xx *_and *S*_*yy *_yields an equation with 16 terms dependent upon the errors terms, (*E*_*x*_(*ω*), *E*_*y*_(*ω*)), the harmonic terms, (*O*_*x*_(*ω*), *O*_*y*_(*ω*)), the transfer functions, *H*_*xx*_, *H*_*xy*_, *H*_*yx*_, *H*_*yy*_, and their complex conjugates. For the *X *channel, these components are as follows:(20)

Each element of the spectra of *S*_*xx *_can be thought of as either intrinsic (caused by the *X*_*t*_), causal (caused by *Y*_*t*_) or cross terms (caused by *X*_*t *_and *Y*_*t*_). Thus(21)

where

In the absence of the harmonic oscillators, there are only four terms in the expression for *S*_*xx *_and *S*_*yy*_, and the cross term can be eliminated using the transformation proposed by Geweke [35,36]. Eliminating the cross term is essential for a consistent definition of the frequency domain causality, however the addition of the harmonic terms makes the prospect of removing the cross term rather troublesome. In the harmonic case, it is not possible, in general to eliminate the cross terms by means of transformation due to the presence of *O*_*x *_and *O*_*y *_terms. The oscillation terms *O*_*x*, *y *_are sinusoidal indicating that the Fourier transforms of these functions are delta functions, and the discontinuous nature of the delta functions makes it impossible to find a transformation eliminate all the cross terms. The method we use to eliminate the cross terms is as follows: firstly we apply the approximation of the Geweke transformation:(22)

where *γ*^2 ^is the covariance matrix between *X *and *Y*, S is the variance of either error *E*_*x*_or *E*_*y*_.

The step from Eq. 22 to Eq. 23 suggests that the transfer matrix *H *is an approximate to the true value. This estimate is necessary to ensure that the causality has a consistent definition. The transfer equation is now as follows:(24)

We combined Eq. 18 and Eq. 24 to define *X*(*ω*) as follows:(25)

Where  and . This has the effect of nullifying the cross terms which contain the element of error. Then the cross terms (components of *S*_*cross*_) are reallocated either to *S*_*causal *_or *S*_*intrinsic *_in the following fashion:

After the reallocation of the components of the spectrum the resultant causality is an approximation rather than a precise calculation, yet it can be shown to yield convincing and consistent results. Using these two methods to approximate the spectrum, we have obtained the spectrum in such a format  as the  term is negligible. In the case where the harmonic term is not present, the causality is defined as:(26)

Yet, it is essential that the causality in the harmonic case is defined in terms of both *S*_*intrinsic *_and *S*_*causal*_. Therefore, by analogy to the normal frequency domain causality (without harmonic terms), the frequency domain causality in the harmonic case is defined as:(27)

In the Results section, we will show through examples that this approximation produces an excellent estimate of the frequency domain causality.

## Authors' contributions

JHW carried out model development and data analysis and wrote up the manuscript. JLS carried out the model development and data analysis. VBW provided the leaf gene data. JFF conceived of the study, participated in design, supervised the studies. All authors read and approved the final manuscript.

## Supplementary Material

Additional file 1 **Supplementary material**. This file contains the Granger causality analysis. Click here for file
