# Supplementary material for: Impact of environmental inputs on reverse-engineering approach to network structures
Source: BMC Syst Biol. 2009 Dec 4;3:113. doi: 10.1186/1752-0509-3-113 (PMC2799448; doi:10.1186/1752-0509-3-113)
Supplement: Additional file 1 — Supplementary material. This file contains the Granger causality analysis. [file 1752-0509-3-113-S1.PDF]

Supplementary Material

It is necessary to recall the more usual form of Granger analysis (without the harmonic term). Consider two time series, X_t and Y_t; the essence of Granger causality is to determine the extent to which the activity of one time series affects the other. It is assumed that the two time series are stationary processes and can be expressed in autoregressive representation: In equations Eqn.28 and Eqn.29, X_t is estimated using only previous information of X, similarly Y_t use only previous components of Y to estimate the value at time t.

$$X_t = \sum_{j=1}^{\infty} a_{1j} X_{t-j} + \epsilon_{1t} \tag{28}$$

$$Y_t = \sum_{j=1}^{\infty} d_{1j} Y_{t-j} + \eta_{1t} \tag{29}$$

With $var(\epsilon_{1t}) = \Sigma_1$ and $var(\eta 1t) = \Gamma_1$.

In contrast, equations Eqn.30 and Eqn.31 show how the time series X_t and Y_t are estimated using previous components of both time series.

$$X_t = \sum_{j=1}^{\infty} a_{2j} X_{t-j} + \sum_{j=1}^{\infty} b_{2j} Y_{t-j} + \epsilon_{2t} \tag{30}$$

$$Y_t = \sum_{j=1}^{\infty} c_{2j} X_{t-j} + \sum_{j=1}^{\infty} d_{2j} Y_{t-j} + \eta_{2t} \tag{31}$$

With $var(\epsilon_{2t}) = \Sigma_2$ and $var(\eta 2t) = \Gamma_2$.

One can infer that there is causal relationship from $X \rightarrow Y$ if the inclusion of the $\sum_{j=1}^{\infty} c_{2j} X_{t-j}$ term in equation 31 reduces the error of the estimation of Y_t, in other words there is a causal relationship from $X \rightarrow Y$ if $\Gamma_2 < \Gamma_1$. Similarly, there is casual link from $Y \rightarrow X$ if $\Sigma_2 < \Sigma_1$. The reduction of the variance of the error is the essence of the Granger causality in the time domain yet the real power of this technique becomes apparent when one considers the frequency domain. There are many examples of time series which exhibit highly periodic behaviour, for example neural local field potentials and gene cycles [17–21], physiological data and financial data. For this reason it is suggested that the time series data might be better approximated using a harmonic term in addition to the autoregressive terms.
